# Early assessment of maternal bleeding: development and validation of a prognostic model predicting death and near misss

**DOI:** 10.1016/S2214-109X(25)00488-7

**Published:** 2026-03-01

**Authors:** Francois-Xavier Ageron, Katharine Ker, Danielle Prowse, Ewout W Steyerberg, Eni Balogun, Haleema Shakur-Still, Folasade Adenike Bello, Hugo Madar, Catherine Deneux-Tharaux, Rizwana Chaudhri, Oladapo Olayemi, Projestine Muganyizi, Loïc Sentilhes, Ian Roberts, for the Anti-fibrinolytics Trialists Collaborators Obstetric Group, Francois-Xavier Ageron, Homa Ahmadzia, Monica Arribas, Eni Balogun, Amy Brenner, Amber Geer, Katharine Ker, Raoul Mansukhani, Danielle Prowse, Ian Roberts, Haleema Shakur-Still, Rizwana Chaudhri, Catherine Deneux-Tharaux, Hugo Madar, Loïc Sentilhes, Oladapo Olayemi, Luis D Pacheco, George Saade

**Affiliations:** Clinical Trials Unit, London School of Hygiene & Tropical Medicine, London, UK; Department of Emergency Medicine, Lausanne University Hospital, University of Lausanne, Lausanne, Switzerland; Clinical Trials Unit, London School of Hygiene & Tropical Medicine, London, UK; Clinical Trials Unit, London School of Hygiene & Tropical Medicine, London, UK; Julius Center, University Medical Center Utrecht, Utrecht, Netherlands; Clinical Trials Unit, London School of Hygiene & Tropical Medicine, London, UK; Clinical Trials Unit, London School of Hygiene & Tropical Medicine, London, UK; Department of Obstetrics and Gynaecology, College of Medicine Ibadan, University of Ibadan, Ibadan, Nigeria; Department of Obstetrics and Gynecology, Bordeaux University Hospital, Bordeaux, France; Obstetrical, Perinatal, and Pediatric Epidemiology Research Team, INSERM, INRAE, Paris, France; Global Institute of Human Development, Shifa Tameer-e-Millat University, Islamabad, Pakistan; Department of Obstetrics and Gynaecology, College of Medicine Ibadan, University of Ibadan, Ibadan, Nigeria; Department of Obstetrics and Gynaecology, Muhimbili University of Health and Allied Sciences, Dar es Salaam, Tanzania; Department of Obstetrics and Gynecology, Bordeaux University Hospital, Bordeaux, France; Clinical Trials Unit, London School of Hygiene & Tropical Medicine, London, UK; Department of Emergency Medicine, Lausanne University Hospital, University of Lausanne, Lausanne, Switzerland; Inova Health System, Falls Church, VA, USA; Clinical Trials Unit, London School of Hygiene & Tropical Medicine, London, UK; Global Institute of Human Development, Shifa Tameer-e-Millat University, Islamabad, Pakistan; Obstetrical, Perinatal, and Pediatric Epidemiology Research Team, Centre for Research in Epidemiology and Statistics, U1153, INSERM, INRAE, Université Paris Cité, Paris, France; Department of Obstetrics and Gynecology, Bordeaux University Hospital, Bordeaux, France; Department of Obstetrics and Gynaecology, College of Medicine, University of Ibadan, Ibadan, Nigeria; Division of Maternal-Fetal Medicine, The University of Texas Medical Branch at Galveston, Galveston, TX, USA; Department of Obstetrics and Gynecology, Eastern Virginia Medical School, Norfolk, VI, USA

## Abstract

**Background:**

Bleeding after childbirth is a leading cause of mortality. Most deaths are in low-income and middle-income countries. Early identification of women at high risk of life-threatening bleeding is vital to guide the use of life-saving interventions. The aim of this study was to develop and validate a prognostic model to predict death and near miss from bleeding after childbirth and to use it to a create simple clinical score to identify women’s level of risk at admission.

**Methods:**

This observational prognostic study used multilevel logistic regression with data from 22 low-income, middle-income, and high-income countries. We used individual patient data from four randomised controlled trials (WOMAN, WOMAN-2, TRAAP, and TRAAP2 trials) that assessed the effect of tranexamic acid administration on maternal death and blood loss after birth, vaginally or by caesarean. The predicted outcome was death or near miss based on the WHO near miss approach to life-threatening postpartum bleeding. Maternal death included all deaths within 24 h of birth. Near miss included laparotomy, hysterectomy, or interventional radiology within 24 h of birth. The model included a random effect for country and trial and underwent internal–external cross-validation. A simple score for clinical use was developed from the coefficient of the regression equation, and the clinical value of the prognostic model and score was assessed by decision curve analysis.

**Findings:**

We included data on 43 450 women enrolled in the four trials. Death or near miss occurred in 1291 women (3·0%). The model predicting death or near miss included age, baseline systolic blood pressure, pre-birth anaemia, caesarean birth, placental disorders, hypertensive disease during pregnancy, and whether the birth being studied was a stillbirth. By use of internal–external cross-validation, the pooled area under the receiving operator curve (AUROC) for the model was 0·82 (95% CI 0·78–0·87). Our simple clinical score to identify women at very low (risk unlikely), low, intermediate, high, or very high risk of death or near miss had an AUROC of 0·80 (0·76–0·84). Discrimination was heterogeneous between countries, with low AUROC (0·58 [0·48–0·68]) in the only high-income country included in the cross-validation (France). The model and the score presented a clinical value with a positive net benefit for a wide range of risk of death or near miss.

**Interpretation:**

We developed a prognostic model and a simple score that can be used at hospital admission to quantify the risk of life-threatening bleeding in women giving birth. This score can be used to inform the need for early tranexamic acid administration in women giving birth in low-income and middle-income countries.

**Funding:**

The Gates Foundation.

## Introduction

Bleeding after childbirth is a leading cause of maternal mortality worldwide.^1^ Almost all these deaths are in low-income and middle-income countries (LMICs).^2^ Identification of women at high risk of death from bleeding after childbirth could improve outcomes by facilitating early intervention. Postpartum haemorrhage is traditionally defined as a blood loss of 500 mL or more from the genital tract within 24 h of vaginal birth.^3^ However, for many women, treatment of established postpartum haemorrhage is too late to prevent death. Most deaths occur in the first 24 h after birth.^4–6^ Furthermore, women with anaemia, who have reduced capacity to tolerate bleeding, can become seriously ill, even if they lose less than 500 mL of blood.^7^

Several studies have sought to develop postpartum haemorrhage prediction tools.^8^ Most aimed to predict blood transfusion or bleeding over a particular blood loss threshold, ranging from 500 mL to 2000 mL.^9,10^ However, all of these prediction models had a high risk of bias and none were suitable for clinical use.^8,9^ Absence of clear target setting, precise timing of model use, and unclear measurement of predictors were the most common biases in the numerous models developed.^8^ Inappropriate methods of analysis or omission of statistical analysis, such as calibration assessment, external validation, and handling of missing data, were found in most of the reported studies.^9^ Blood transfusion is an intervention rather than a health outcome and transfusion practice varies between settings. Blood loss is more relevant but is prone to measurement error and does not identify women with anaemia, who are at high risk of life-threatening bleeding.^11^ There are no prediction models for maternal death from bleeding after childbirth.^5,8^ To reduce deaths from postpartum haemorrhage, a risk stratification model for the risk of life-threatening bleeding needs to be developed.^6^ We developed and validated a prognostic model to predict death and near miss from bleeding after childbirth. The authors use women to describe people who have experienced pregnancy and childbirth, but do not intend to exclude people who might use different terms.

## Methods

### Study population

We used individual patient data from four large randomised controlled trials assessing the effect of tranexamic acid administration on blood loss after birth (WOMAN [2017], WOMAN-2 [2024], TRAAP [2018], and TRAAP2 [2021] trials).^12–15^ The study data are described in detail elsewhere.^16^ Individual patient data were checked for consistency and completeness by one author (KK) and the risk of bias was assessed by the Cochrane risk-of-bias tool.^17^ All trials were prospectively registered and included 500 or more participants. The trials included women giving birth vaginally or by caesarean. We selected trials of tranexamic acid because they included participants from prevention and treatment trials and the individual patient data were available.^17^ Inclusion and exclusion criteria are listed in appendix 1 (pp 1–2). Of note, TRAAP and TRAAP2 trials excluded women with placenta previa or placenta accreta. The TRAAP2 trial excluded women with severe prenatal anaemia (ie, haemoglobin ≤90 g/L). Institutional review board approval for this study was not required. Each trial included in this study had previously received all the appropriate ethical approvals. This study did not include identifiable data.

### Outcome

Our objective was to predict death or near miss due to bleeding. We used all-cause mortality within 24 h of birth, because later deaths are unlikely to be due to bleeding. We used the near miss criteria defined by WHO (ie, interventional radiology or laparotomy [including hysterectomy but excluding caesarean] within 24 h of birth). We excluded massive transfusion and intensive care unit admission because these interventions are often unavailable in low-income and middle-income settings.^18^

### Predictors and variable selection

We included in the model covariables that we expected to be associated with the outcome and that were available at the time of birth. We were careful not to include variables on the causal pathway between another variable and the outcome to avoid overadjustment and collider bias. We used a directed acyclic graph to identify potential covariables (appendix 1 p 3). We included age, BMI, baseline haemoglobin (used to define anaemia severity as severe [haemoglobin <70 g/L], moderate [haemoglobin 70–99 g/L] or mild [haemoglobin 100–109 g/L]), baseline systolic blood pressure, placenta abnormalities (ie, placenta praevia, placenta accreta, abruption, or polyhydramnios), parity, previous caesarean, type of birth (ie, vaginal or caesarean birth), previous postpartum haemorrhage, diabetes during pregnancy, hypertensive disease during pregnancy (including pre-eclampsia), stillbirth (ie, whether the birth being studied was a stillbirth), country income category defined by the World Bank, and trial treatment group (tranexamic acid or placebo). Baseline haemoglobin and systolic blood pressure refers to measurements taken at the time of randomisation. Randomisation was at hospital admission in the TRAAP, TRAAP2, and WOMAN-2 trials. For the WOMAN trial, randomisation was after the diagnosis of postpartum haemorrhage.

### Model development

We used multilevel logistic regression with a random effect for the country and the trial. We used a backward stepwise method including all variables in the original regression equation and then removed one variable at a time based on the p value of the Wald test (ie, if p>0·05).^19^ We also used the least absolute shrinkage and selection operator (LASSO) method as a sensitivity analysis to control for variable selection.^20^ We used a cross-validation model to select the optimal lambda value (λ). We determined the minimum λ value and the standard error of the cross-validation for each λ. We selected the λ as one standard error. Because we used a moderate number of predictors, we chose the backward stepwise method to manually control the inclusion of predictors and to avoid coefficient estimates bias due to the shrinkage method. All continuous variables were included in the model as linear terms. We graphically assessed their relationship with the outcome. If log-linearity was reasonable, we included quadratic or cubic terms or a cubic spline in addition to the linear term in the equation. We tested for plausible interactions between age, baseline systolic blood pressure, type of birth, placenta abnormality, and anaemia. We assessed calibration as the difference between the observed and predicted probabilities. We plotted observed and predicted probabilities by decile of risk and with a non-parametric smooth function.

### Model validation

We conducted an internal–external cross-validation procedure.^21^ We selected countries with a sample size of more than 800 women and followed all model development steps in all the countries except one. We estimated the discrimination with the area under the receiving operator curve (AUROC) and the calibration as the ratio of the predicted and observed probabilities (E/O) in the remaining country. The AUROCs and the ratio E/O for each country were pooled with random effects meta-analysis methods.^22^ We plotted observed and predicted probabilities by decile of risk and with a non-parametric smooth function for each country.

### Derivation and validation of a clinical score

We derived a simple score for clinical use, which we named the bleeding assessment for maternal death or near miss (BAD) score. For each predictor, we assigned an integer value that was proportional to the coefficient of the regression equation of the final prognostic model. We estimated the sensitivity, specificity, and likelihood ratio for each category of the score to assess accuracy. We defined different categories of risk of death or near miss: risk unlikely (when the risk of death or near miss was <1%), low risk (1–4%), intermediate risk (5–9%), high risk (10–19%), and very high risk (≥20%). We estimated the AUROC of the score for each country in the cross-validation procedure. We pooled the AUROCs with random effects meta-analysis methods.

### Clinical use and decision making

We also assessed the clinical value of the prognostic model and score. We plotted the decision curve analysis as described by Vickers aand Elkin.^23^ The decision curve analysis plots the net benefit on the threshold probabilities of the predicted risk of death or near miss. The net benefit represents the difference between the benefit of treating the true positive and the harm of treating the false positive:

$$\text{Net}\;\text{benefit}\;=\frac{\;\text{True}\;\text{positive}\;}{\text{N}}-\frac{\;\text{False}\;\text{positive}\;}{\text{N}}\left(\frac{\text{Pt}}{1-\text{Pt}}\right)$$

where Pt is the threshold probability. The prediction model and score have a clinical value in decision making when the net benefit of the model is superior to a strategy of treating all women. For the clinical score, we estimated the threshold probabilities with a logistic calibration method and compared the net benefit of the score with the net benefit of the prognostic model. We performed a cluster analysis using the k-means algorithm to identify clinical scenarios associated with baseline risk stratification.

### Sample size and missing data

The sample size was fixed. All trials included in this study had a low proportion of missing variables (<1%), except for baseline haemoglobin (2·2%). Some predictors were not collected in all trials. Baseline haemoglobin, diabetes during pregnancy, hypertensive disease, and BMI were not collected in the WOMAN trial. We considered that these variables were missing completely at random and that a complete case analysis could be considered unbiased. To deal with the statistical power reduction due to complete case analysis, we included the value unknown in the regression equation for diabetes during pregnancy and hypertensive disease when a trial did not collect them. To estimate model performance, we replaced the coefficient for unknown to be the null value. For baseline haemoglobin and BMI, which were missing in the WOMAN trial, we used multiple imputation with chained equations because the setting and clinical situations in the WOMAN and WOMAN-2 trials were similar.^24^ We considered that these variables were missing at random and that multiple imputation would be less biased than a complete case analysis.^25^ Independent variables used in the multiple imputation with chained equations were death within 24 h, laparotomy, hysterectomy, country, age, income, baseline systolic blood pressure, blood transfusion, and haemodynamic instability (defined by hypotension or tachycardia). We generated 20 imputed datasets. All analyses were performed with Stata software version 18.0. LASSO analysis was performed with R package Glmnet version 4.1-4.

### Role of the funding source

The funder of the study had no role in study design, data collection, data analysis, data interpretation, or writing of the report.

## Results

We included individual patient data on 43 450 women from four randomised controlled trials. The WOMAN and WOMAN-2 trials mostly included women giving birth in LMICs (596 [2·8%] of 20 060 participants in the WOMAN trial were from the UK [a high-income country]; all other women included in the WOMAN and WOMAN-2 trials were from LMICs), and the TRAAP and TRAAP2 trials only included women from France, a high-income country (table 1). Women in the WOMAN and WOMAN-2 trials were younger than those in the TRAAP and TRAAP2 trials. Baseline characteristics varied between trials, depending on the inclusion criteria: the WOMAN trial included women with a diagnosis of postpartum haemorrhage; the WOMAN-2 trial included women with moderate or severe anaemia who were giving birth vaginally; the TRAAP trial included women who had a singleton pregnancy who were giving birth vaginally; and the TRAAP2 trial included women with a singleton or multiple pregnancy expected to undergo caesarean birth. When predictors were collected, missing values were 2·2% (524/23390). Missing values for potential predictors in each trial are shown in appendix 1 (p 4). Death or near miss occurred in 1291 women (3·0%).

**Figure 1:**
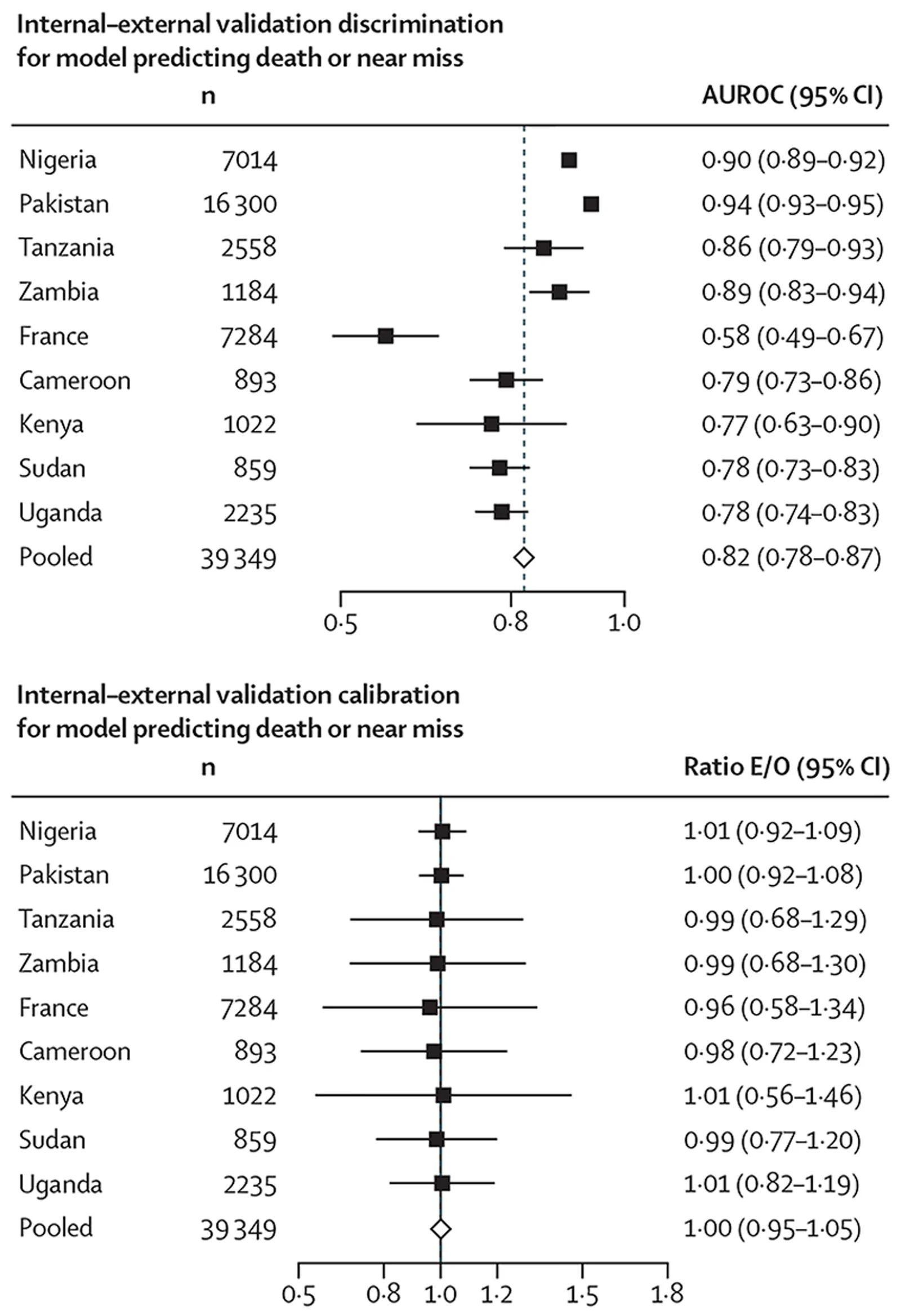
Internal–external cross-validation for discrimination and calibration of the BAD prognostic model Internal−external cross-validation only included countries with a sample size of more than 800 women. Some CIs for Nigeria and Pakistan are too small to be visualised, and so those error bars are not included in the plot. AUROC=area under the receiving operator curve. BAD=bleeding assessment for maternal death or near miss. E/O=ratio of the predicted and observed probabilities.

The final model predicting death or near miss included age, baseline systolic blood pressure, anaemia severity, caesarean, placenta abnormality, hypertensive disease during pregnancy, and stillbirth (appendix 1 p 5). The LASSO found a similar variable selection as the backward stepwise selection (appendix 1 p 6). The BAD score was developed from the coefficient of the regression equation (table 2). At internal–external cross-validation, pooled AUROC for the full model was 0·82 (95% CI 0·78–0·87) with an outlier for France (0·58 [0·49–0·67]; figure 1). Pooled ratio E/O was 1·00 (0·95–1·05). The calibration curves for each country in the cross-validation are shown in appendix 1 (p 7).

**Figure 2:**
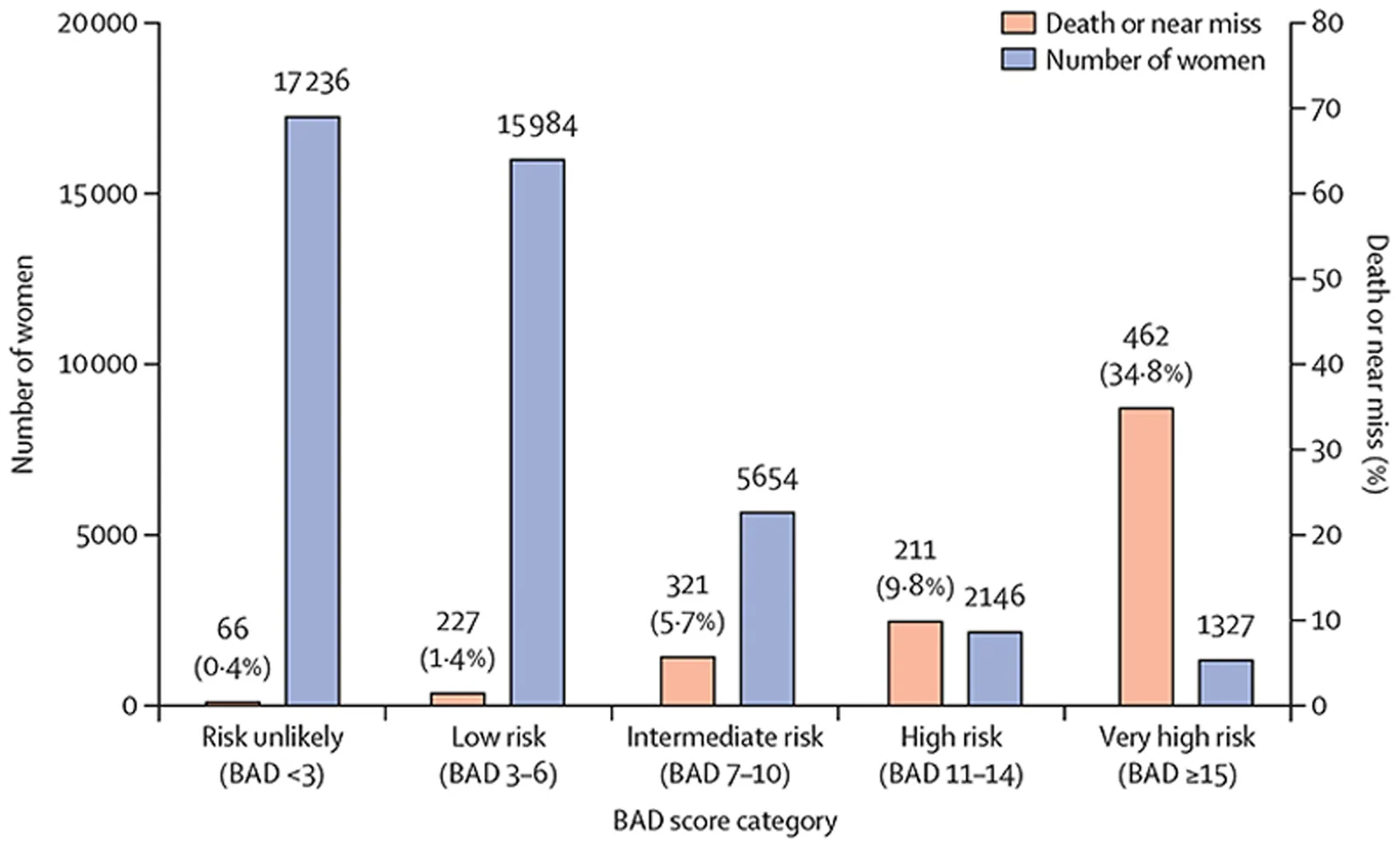
Proportion of death or near miss by BAD score risk stratification Note that scales on y axes differ between plots. BAD=bleeding assessment for maternal death or near miss.

**Figure 3:**
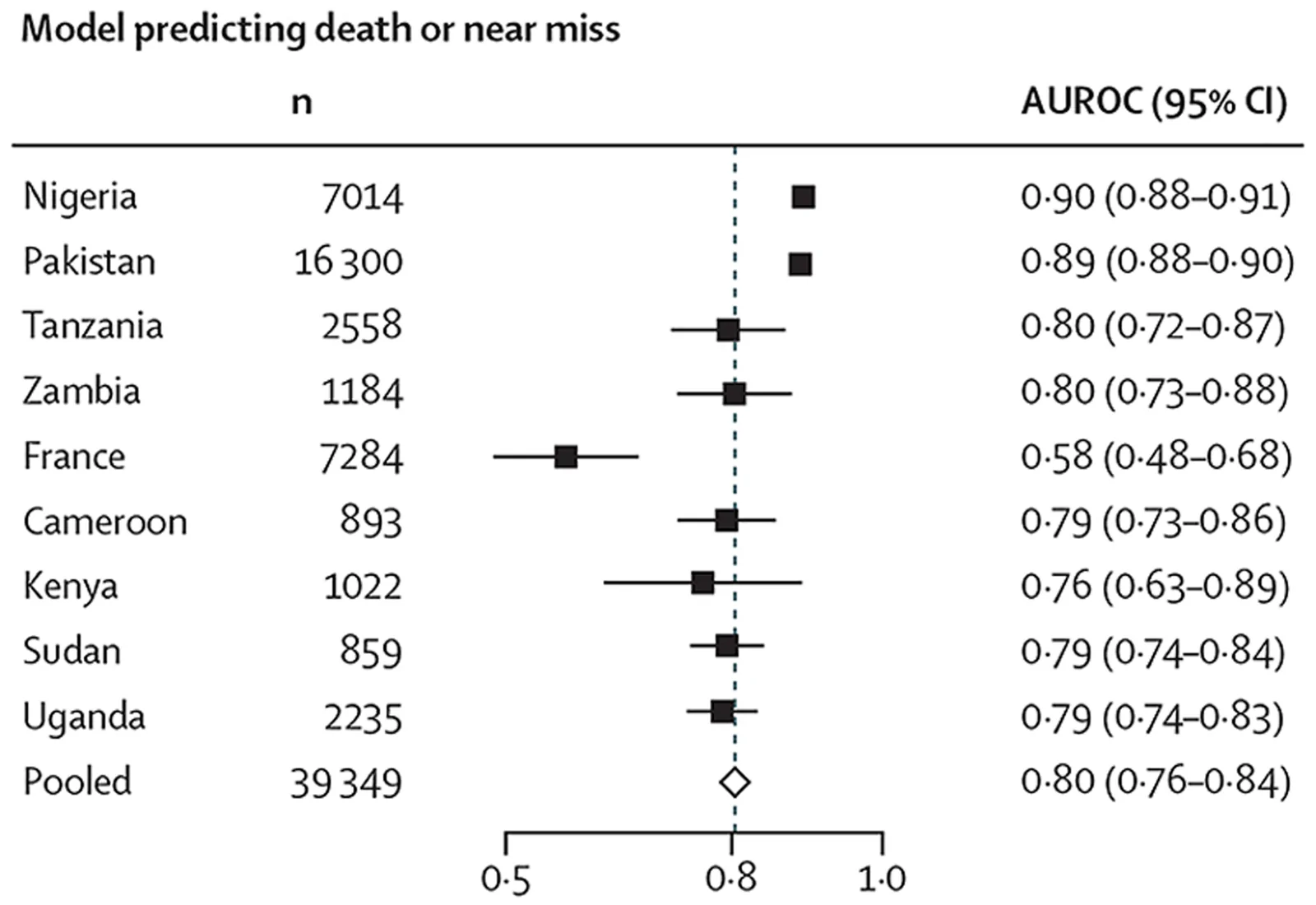
Internal–external cross-validation for discrimination of the BAD score Internal−external cross-validation only included countries with a sample size of more than 800 women. CIs for Nigeria and Pakistan are too small to be visualised, and so those error bars are not included in the plot. AUROC=area under the receiving operator curve. BAD=bleeding assessment for maternal death or near miss.

**Figure 4:**
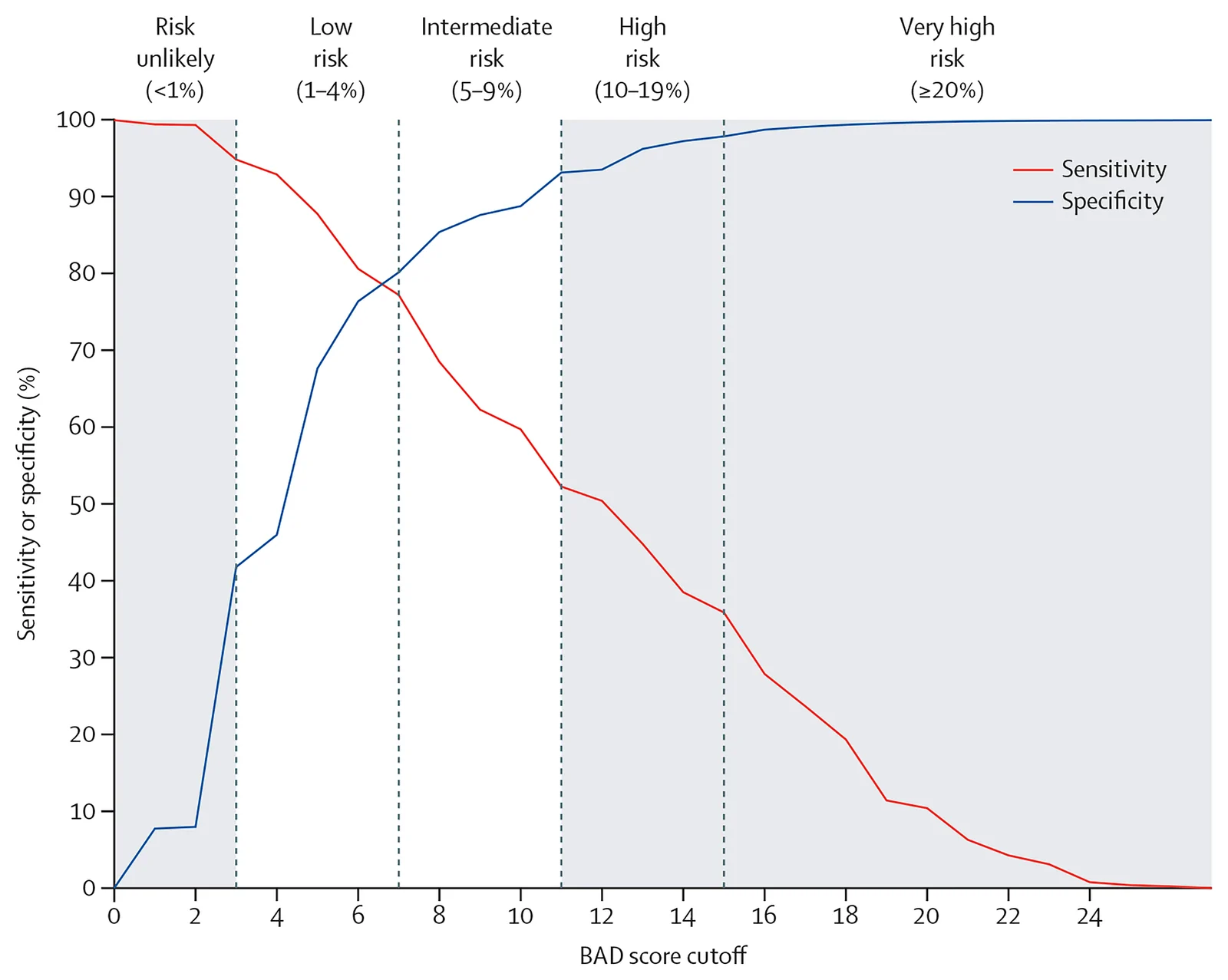
Sensitivity–specificity rule-in and rule-out for BAD score Percentages in parentheses for risk categories are risk of death or near miss. BAD=bleeding assessment for maternal death or near miss.

We defined BAD categories according to the risk of death and near miss: risk unlikely (BAD <3), low risk (BAD 3–6), intermediate risk (BAD 7–10), high risk (BAD 11–14), and very high risk (BAD ≥15). Across BAD score categories, the risk of death and near miss increased from 0·4% (66/17 236 women in risk unlikely category) to 34·8% (462/1327 women in very high-risk category; figure 2). The pooled AUROC for the BAD score was 0·80 (95% CI 0·76–0·84; figure 3). The sensitivity of the BAD score was 95% for the low-risk category and the specificity of the BAD score was 93% for the high-risk category (figure 4). The decision curve analysis showed a net benefit from use of the prognostic model compared with a treat-all-women strategy (appendix 1 p 8). The net benefit for the BAD score was slightly inferior to the prognostic model.

We show potential clinical scenarios for women in each BAD score category based on age, anaemia severity, hypotension, and other risk factors (caesarean, hypertensive disease, and placenta abnormalities; appendix 1 p 10). Women younger than 30 years with no risk factors have a BAD score of 0, indicating that the risk of death or near miss is low; therefore, no specific intervention, such as tranexamic acid, is required. Women older than 30 years who have given birth by caesarean section and have additional risk factors have a BAD score greater than 4, suggesting an intermediate risk; these women should receive tranexamic acid. Women with haemodynamic instability are at high risk of death or near miss from postpartum haemorrhage and should also be treated with tranexamic acid.

## Discussion

We developed a simple score that can be used at hospital admission to quantify the risk of life-threatening bleeding in women giving birth. Our score can inform the need for tranexamic acid administration at the time of birth in low-income and middle-income countries. Results from a recent individual participant data meta-analysis^17^ show that early tranexamic acid use reduces the risk of life-threatening bleeding (odds ratio 0·77, 95% CI 0·63–0·93). Because the absolute benefit and cost-effectiveness of tranexamic acid administration is greatest in women at high risk, an accurate method to identify such women is urgently needed. We found that age, baseline systolic blood pressure, anaemia severity, caesarean birth, hypertensive disease, placenta abnormalities, and stillbirth are the main predictors. Because women with moderate or severe anaemia can become shocked despite moderate blood loss, the inclusion of anaemia severity in our model is important, especially in sub-Saharan Africa and south Asia, where most pregnant women have anaemia.^26^ Although the accuracy of our model in high-income countries is open to question and requires further research, the benefits of tranexamic acid administration at the time of birth are also much less clear in these settings. All women with established postpartum haemorrhage should receive tranexamic acid as per WHO guidelines. In women at high risk, the balance of benefits and possible harms is clearly favourable and tranexamic acid treatment at the time of birth is likely to be effective and cost-effective.

Our study has many strengths. Because we used data from large high-quality trials, our model was developed in a well described inception cohort with almost complete follow-up and minimal missing data. We also used well defined outcomes that were assessed at a fixed timepoint. Although we included women from randomised controlled trials of tranexamic acid for the prophylaxis and treatment of severe bleeding, all prognostic factors were collected before the outcome (death or near miss). All trials had clear definitions of prognostic factors and outcomes, with standard operating procedures for data collection and quality control procedures that minimise systematic errors in measurements, thus helping to ensure high quality. The inclusion of women from LMICs—where most maternal haemorrhage deaths occur—increases the relevance of our results. Developing robust models requires many outcome events. For this reason, we did not split the study population into derivation and validation cohorts but instead performed an internal–external cross-validation to increase statistical power.^21^ Combining individual patient data from different randomised controlled trials done in different settings increases the sample size and the heterogeneity of the data, which reduces overfitting and increases generalisability.^27,28^ The use of LASSO to assess covariate selection should also reduce overfitting.^28,29^ We used a multilevel model with random effect for country and trial to account for heterogeneity between trials.^30^ Heterogeneity is more likely to increase the generalisability of the model in the setting where the model performed well. The internal–external cross-validation procedure allowed us to assess how well the model performs in different settings. Further external validation is needed in high-income settings, and we welcome external validation by different authors.

Our study also has limitations. The use of data from clinical trials to develop a prognostic model can be a limitation if the trials have strict inclusion criteria, which might limit the generalisability of the results. However, in this case, we included data from large pragmatic trials with broad eligibility criteria. Measurement error for variables such as baseline systolic blood pressure and haemoglobin concentration might have introduced regression dilution bias, which would underestimate the effect of the predictor by flattening the regression line.^31^ Some measurement error is unavoidable in clinical practice and can be larger outside of trial contexts. Random error also increases the standard error of the regression coefficients, thus reducing the precision of the estimate. Some of the outcomes could also have been misclassified despite the high standard of the included trials.

Although the heterogeneity of our data helps to reduce overfitting and increase generalisability, it also brings limitations. Data harmonisation can be difficult and could have resulted in differences in the measurement of risk factors. Combining data from prophylactic and therapeutic trials might also have introduced bias. Patients in the WOMAN trial had a diagnosis of postpartum haemorrhage before randomisation and so were more likely to have hypotension. We tested multiple interactions in the model and included a random effect by trial in the multilevel model to account for variability. We included the interaction between type of birth and placenta abnormalities in the final model. We assessed collinearity by estimating variance inflation factors and found no obvious collinearity. There were few missing data, but if the missing values were not missing completely at random, then our use of multiple imputation might be biased.^25^ Haemoglobin concentration was not collected in the WOMAN trial. We therefore used multiple imputation based on the data from prophylactic trials, but we acknowledge that this method could have increased the importance of anaemia in this model. To ensure internal validity, we assessed the association between anaemia and death or near miss without multiple imputation corresponding to a stratified analysis in addition to the multilevel model.

Our model did not fit well in France, the only high-income country included in the cross-validation. The low numbers of death or near miss outcome events might account for the poor fit. Although this does not necessarily mean that the identified prognostic factors are unimportant in high-income settings, further studies in high-income countries are needed.

Our model can be used to improve patient outcomes in low-income and middle-income settings. Because our clinical score is based on information available at hospital admission, it allows rapid risk stratification and early intervention. Our model could also be used for audit and feedback. For example, the proportion of women at high risk who received tranexamic acid or other indicated treatments could be audited to improve patient care.

## Supplementary Material

Supplementary File: Prognostic

The Antifibrinolytics Trialists Collaborators Obstetric Group

## Figures and Tables

**Table 1:** Characteristics of the study participants

	WOMAN (n= 20 060)	WOMAN-2 (n=15 068)	TRAAP (n=3891)	TRAAP2 (n=4431)
Setting	LMICs	LMICs	HIC	HIC
Type of birth	Vaginal and caesarean	Vaginal	Vaginal	Caesarean
Mean age (SD), years	28·3 (5·7)	27·2 (5·6)	30·2 (4·9)	33·4 (5·3)
Age ≥30 years	8603 (42·9%)	5236 (34·7%)	2184 (56·1%)	3313 (74·8%)
Diagnosis of postpartum haemorrhage at baseline	Yes	No	No	No
Mean BMI (SD), kg/m^2^	NA	26·5 (3·8)	23·4 (4·5)	26·2 (6·3)
Placenta abnormalities*	1885 (9·4%)	555 (3·7%)	33 (0·8%)	83 (1·9%)
Multiple pregnancy	1042 (5·2%)	583 (3·9%)	0	319 (7·2%)
Mean baseline haemoglobin (SD), g/L	NA	82·8 (11.8)	117·9 (10·2)	118·0 (11·0)
Mild anaemia (haemoglobin 100–109 g/L)	NA	0	583 (15·0%)	718 (16·2%)
Moderate anaemia (haemoglobin 70–99 g/L)	NA	12989 (86·2%)	141 (3·6%)	210 (4·7%)
Severe anaemia (haemoglobin <70 g/L)	NA	2079 (13·8%)	0	0
Stillbirth	1986 (9·9%)	1036 (6·9%)	0	0
Hypertensive disease during pregnancy	NA	1146 (7·6%)	84 (2·2%)	283 (6·4%)
Diabetes during pregnancy	NA	89 (0·6%)	420 (10·8%)	946 (21·3%)
Death within 30 days	483 (2·4%)	18 (0·1%)	0	0
Death within 24 h	355 (1·8%)	11 (0·1%)	0	0
Near miss†	995 (5·0%)	27 (0·2%)	8 (0·2%)	20 (0·5%)

Hutcheon JA, Chiolero A, Hanley JA. Random measurement error and regression dilution bias. *BMJ* 2010; **340:** c2289.Data are n (%), unless otherwise specified. HIC=high-income country. LMIC=lower-income and middle-income country. NA=not available.

* Placenta abnormalities included placenta praevia, placenta accreta, abruption, or polyhydramnios.

† Near miss included laparotomy, hysterectomy, or interventional radiology within 24 h of delivery.

**Table 2:** BAD model

	OR (95% CI)	Point
Age		
30–39 years	1.8 (1·5–2·0)	2
≥40 years	2·4 (1·8–3·1)	3
Baseline systolic blood pressure		
Mild hypotension (80–100 mm Hg)	2·5 (2·2–2·9)	3
Severe hypotension (<80 mm Hg)	7·5 (6·2–8·9)	8
Caesarean	2·4 (2·1–2·8)	2
Placenta abnormalities*	1·4 (1·0–2·0)	1
Hypertensive disease during pregnancy	2·4 (1·8–3·3)	2
Anaemia severity		
Moderate anaemia (haemoglobin 70–99 g/L)	2·7 (1·3–5·8)	2
Severe anaemia (haemoglobin <70 g/L)	22·6 (10·4–48·8)	10
Stillbirth	3·0 (2·5–3·5)	3

The full model included interaction between type of birth and placenta abnormalities (p=0·01) and tranexamic acid (OR 0·85, 95% CI 0·75–0·96). BAD=bleeding assessment for death or near miss. OR=odds ratio.

* Placenta abnormalities included placenta praevia, placenta accreta, abruption, or polyhydramnios.

## Data Availability

Anonymised data from the WOMAN trial are available for download from the FreeBIRD data repository. Anonymised data from the WOMAN-2 will also be available from FreeBIRD after publication of the primary and secondary trial analyses. Request for data from the TRAAP trials can be made to the trials’ steering committees via the lead investigators.

## Research in context

### Evidence before this study

Postpartum haemorrhage is usually defined as blood loss of 500 mL or more from the genital tract within 24 h of vaginal birth. Several studies have sought to develop prediction tools for postpartum haemorrhage. Most attempted to predict blood transfusion or bleeding beyond a particular blood loss threshold ranging from 500 mL to 2000 mL. Blood transfusion is an intervention rather than a health outcome and transfusion practice varies over time and between settings. Blood loss is more relevant but is prone to measurement error and does not identify women with anaemia who are at high risk of life-threatening bleeding. After a systematic literature searches in MEDLINE and Embase databases for articles published between Jan 1, 1946, to March 21, 2025, with the search terms “postpartum”, “birth”, “childbirth”, “caesarean”, “vaginal”, or “maternal” and “hemorrhage”, “bleed”, or “blood” and “prognosis”, “model”, or “statistical”, we found no prediction models for death or near miss after childbirth.

### Added value of this study

We developed and validated a prognostic model to predict early death and near miss from bleeding in low-income and middle-income countries. We developed our model using high-quality data from four randomised controlled trials including 43 450 women. Our model and the related simple score for use at the bedside (bleeding assessment death or maternal near miss score) showed excellent discrimination in low-income and middle-income countries; was able to stratify women based on their risk of death or near miss; and can identify women who require specific intervention such as tranexamic acid administration.

### Implications of all the available evidence

Our model accurately predicts death or near miss from bleeding after birth in low-income and middle-income settings. As the model allows rapid risk stratification, it can guide early intervention. For many postpartum haemorrhage interventions, including tranexamic acid, the proportional reduction in risk is similar regardless of baseline risk. However, the absolute benefit from treatment will be greatest in women who are at high risk of postpartum haemorrhage. By identifying these women, our model helps to ensure that the balance of benefits and harms is most clearly favourable and maximally cost-effective.
