# Supplementary material for: Early assessment of maternal bleeding: development and validation of a prognostic model predicting death and near misss
Source: Lancet Glob Health. Author manuscript; Available in PMC 2026 Oct 30. (PMC7619660; doi:10.1016/S2214-109X(25)00488-7)
Supplement: Supplementary file 1 [file EMS218566-supplement-Supplementary_File__Prognostic.docx]

**Appendix**

**Contents**

[**S1. Inclusion and exclusion criteria**	1](#_Toc219101949)

[**S2. Directed Acyclic Graph (DAG)**	3](#_Toc219101950)

[**S3. Missing for main predictors**	4](#_Toc219101951)

[**S4. Logistic regression equation for predicting early death or near miss**	5](#_Toc219101952)

[**S5. Least Absolute Shrinkage and Selection Operator model for early death and near-miss**	6](#_Toc219101953)

[**S6. Calibration curve by country of the cross-validation procedure**	7](#_Toc219101954)

[**S7. Comparison of the Decision Curve Analysis for the prognostic model and the BAD score**	8](#_Toc219101955)

[**S8. Characteristics of women by BAD risk score stratification**	9](#_Toc219101956)

[**S9. Women clinical picture by baseline risk**	10](#_Toc219101957)

# **S1. Inclusion and exclusion criteria**

| **TRAAP Trial** | |
| --- | --- |
| **Inclusion criteria** | **Exclusion criteria** |
| women 18 years of age or older who had a singleton pregnancy at 35 weeks 0 days of gestation or more and who were planning to undergo vaginal delivery. | Previous episode of venous thrombosis: deep vein thrombosis, pulmonary embolism |
|  | Previous episode of arterial thrombosis: myocardial infarction, stroke |
|  | Any known cardiovascular, kidney, or liver disorder |
|  | Autoimmune disease |
|  | Sickle cell disease |
|  | Condition at possibly increased risk of bleeding: severe hemorrhagic disease, placenta previa, placenta accrete spectrum, HELLP syndrome, multiple pregnancy |
|  | Condition that could impair initial hemostasis: in utero fetal death, administration of low-molecular weight heparin or antiplatelet agents in the week before delivery |
|  | Epilepsy |
|  | Eclampsia |
|  | Poor comprehension of oral French |

1

| **TRAAP-2 trial** | |
| --- | --- |
| **Inclusion criteria** | **Exclusion criteria** |
| women 18 years of age or older with a singleton or multiple pregnancy at 34 or more weeks of gestation and were expected to undergo cesarean delivery before or during labor. | Previous episode of venous thrombosis: deep vein thrombosis, pulmonary embolism |
|  | Previous episode of arterial thrombosis: myocardial infarction, stroke |
|  | Any known cardiovascular, kidney, or liver disorder |
|  | Autoimmune disease |
|  | Sickle cell disease |
|  | Condition at possibly increased risk of bleeding: severe hemorrhagic disease, placenta previa, abnormally invasive placenta, HELLP (Hemolysis Elevated Liver Low Platelet) syndrome. |
|  | Condition that could impair initial hemostasis: in utero fetal death, administration of low-molecular weight heparin or antiplatelet agents in the week before delivery |
|  | Epilepsy or history of epilepsy or seizure |
|  | Eclampsia |
|  | Prenatal hemoglobin level in the week before the cesarean ≤ 9 g/dl |
|  | Known hypersensitivity to tranexamic acid or sodium chloride |
|  | Failed operative vaginal delivery |
|  | Planned general anesthesia |
|  | Cesarean delivery for the second twin or second/third triplet(s) after vaginal birth of the first |
|  | Poor comprehension of oral French |

| **WOMAN Trial** | |
| --- | --- |
| **Inclusion criteria** | **Exclusion criteria** |
| women aged 16 years and older with a clinical diagnosis of post-partum haemorrhage after a vaginal birth or caesarean section  diagnosis of primary post-partum haemorrhage could be based on clinically estimated blood loss of more than 500 mL after vaginal birth or 1000 mL after caesarean section or any blood loss sufficient to compromise haemodynamic stability.  The responsible clinician’s ‘uncertainty’ as to whether or not to use an antifibrinolytic agent in a particular woman with postpartum haemorrhage. | Women for whom the responsible doctor considers there is a clear indication for antifibrinolytic therapy should not be randomised. |
|  | Women for whom there is considered to be a clear contraindication to antifibrinolytic therapy should not be randomised. |
|  | Where the responsible clinician is substantially uncertain as to the appropriateness of antifibrinolytic agents in a particular woman with PPH. |

| **Woman-2 Trial** | |
| --- | --- |
| **Inclusion criteria** | **Exclusion criteria** |
| Women with moderate or severe anaemia (haemoglobin level <30%), who have given birth vaginally and for who the responsible clinician is substantially uncertain whether to use TXA. | Women who are not legally adult (<18 years) and permission not provided by a guardian. |
|  | Women with a known allergy to TXA or its excipients. |
|  | Women who develop PPH before umbilical cord is clamped/cut. |

2

# **S2. Directed Acyclic Graph (DAG)**

PPH: Post-Partum Haemorrhage; BMI: Body Mass Index; SBP: Systolic Blood Pressure; HB: Haemoglobin; APH: Ante-partum haemorrhage

3

# **S3. Missing for main predictors**

|  | N (%) |
| --- | --- |
| Death or near miss | 2 (0·00) |
| Age | 12 (0·03) |
| Body Mass Index   - Except woman trial | 20176 (46·4)  116 (0·5) |
| Placenta abnormalities | 4 (0·01) |
| Type of birth | 5 (0·01) |
| Baseline Haemoglobin   - Except Woman trial | 20584 (47·4)  524 (2·2) |
| Stillbirth | 0 |
| Gestational diabetes   - Except Woman trial | 20064 (46·2)  4 (0·02) |
| Hypertensive disease during pregnancy   - Except Woman trial | 20064 (46·2)  4 (0·02) |
| Multiple pregnancy | 0 |
| Systolic blood pressure | 568 (1·3) |

4

# **S4. Logistic regression equation for predicting early death or near miss**

Predicted probability of death or near miss: $P=\frac{1}{1+ⅇ^{-b}}$

Where b= -6·681413 + random intercept + [0·5647259 if Age=30-39 or 0·8557902 if Age≥ 40] + [2·009289 if SBP<80 or 0·9140522 if SBP=80-99] + [3·116237 if Hb<70 or 0·9975182 if Hb=70-99] + [0·8913966 if c-section and no placenta disease or 1·391642 if c-section and placenta disease] + [0· 3519524 if placenta disease and vaginal birth or 0·8521979 if placenta disease and c-section] + [1·0086145 if stillbirth] + [0·8907399 if hypertensive disease] - 0·1618627 if TXA administration

|  | N | random intercept | Baseline risk (%) |
| --- | --- | --- | --- |
| Nigeria | 7037 | -1·043728 | 6·2 |
| Pakistan | 16307 | -0·587343 | 2·9 |
| Tanzania | 2567 | 0·360970 | 1·5 |
| Zambia | 1184 | -0·065037 | 3·0 |
| France | 8322 | -0·069795 | 0·4 |
| Albania | 485 | -0·157478 | 2·4 |
| Bangladesh | 325 | -0·067240 | 2·9 |
| Burkina Faso | 142 | 0·236147 | 4·0 |
| Cameroon | 893 | 0·456552 | 5·6 |
| Colombia | 8 | 0·843334 | 19·6 |
| Cote d'Ivoire | 8 | -0·098189 | 1·8 |
| Democratic Republic of Congo | 457 | -1·732305 | 1·3 |
| Egypt | 33 | 1·180972 | 9·0 |
| Ethiopia | 302 | 1·047535 | 12·0 |
| Ghana | 41 | 0·632203 | 4·5 |
| Jamaica | 73 | 0·189596 | 3·6 |
| Kenya | 1031 | -0·368151 | 1·9 |
| Nepal | 533 | -0·263604 | 2·4 |
| Papua New Guinea | 38 | 0·583187 | 5·1 |
| Sudan | 860 | 0·396297 | 8·5 |
| Uganda | 2235 | 0·142415 | 4·9 |
| United Kingdom | 569 | -0·107764 | 2·4 |

5

# **S5. Least Absolute Shrinkage and Selection Operator model for early death and near-miss**

|  | Coefficient for λ=0·011168 |
| --- | --- |
| Age | 0·0000325257 |
| Systolic Blood Pressure | 0·0531309091 |
| Anaemia | 0·0179259546 |
| Type of birth | 0·0235123158 |
| Placenta abnormalities | 0·0272253864 |
| Stillbirth | 0·0221438451 |
| Multiparity | - |
| Previous caesarean | - |
| Previous PPH | 0·0007713379 |
| Diabetes | - |
| Hypertensive disease | - |
| Twin pregnancy | - |
| Country income | - |
| Intercept | -0·0080093650 |

**Mean square error by Log(λ) plot**

6

# **S6. Calibration curve by country of the cross-validation procedure**

7

# **S7. Comparison of the Decision Curve Analysis for the prognostic model and the BAD score**

8

# **S8. Characteristics of women by BAD risk score stratification**

|  | Risk unlikely | Low-risk | Intermediate risk | High-risk | Very high-risk |
| --- | --- | --- | --- | --- | --- |
| Age |  |  |  |  |  |
| <30 | 14922 (87) | 5381 (34) | 2504 (44) | 651 (30) | 256 (19) |
| 30-39 | 2314 (13) | 9645 (60) | 2784 (49) | 1344 (63) | 908 (68) |
| ≥40 | 0 | 958 (6) | 366 (7) | 151 (7) | 163 (12) |
| Anaemia |  |  |  |  |  |
| ≥ 110 | 6,378 (37) | 5,608 (35) | 1,457 (26) | 262 (12) | 27 (2) |
| 100-109 | 638 (4) | 514 (3) | 77 (1) | 7 (0) | 0 |
| 70-99 | 10220 (59) | 9862 (62) | 3054 (54) | 409 (19) | 106 (8) |
| <70 | 0 | 0 | 1066 (19) | 1468 (69) | 1194 (90) |
| Caesarean section | 1256 (7) | 5353 (34) | 2109 (37) | 528 (25) | 569 (43) |
| Placenta abnormalities | 95 (1) | 935 (6) | 771 (14) | 310 (15) | 424 (32) |
| Hypertensive disease | 159 (1) | 1123 (7) | 392 (7) | 132 (6) | 123 (9) |
| Stillbirth | 0 | 864 (5) | 1176 (22) | 362 (17) | 617 (47) |
| Systolic blood Pressure |  |  |  |  |  |
| >100 | 17236 (100) | 12419 (78) | 2184 (39) | 1315 (61) | 404 (30) |
| 80-99 | 0 | 3565 (22) | 2647 (47) | 272 (13) | 535 (40) |
| <80 | 0 | 0 | 823 (15) | 559 (26) | 388 (29) |
| Low- and middle-income country | 13171 (76) | 1,689 (79) | 5199 (92) | 2113 (98) | 1327 (100) |

Results are based on multiple imputation data.

9

# **S9. Women clinical picture by baseline risk**

**RISK FACTORS**

- Hypertensive disease during pregnancy (including pre-eclampsia)
- Placenta abnormalities (Placenta Praevia, Placenta Accreta, Polyhydramnios, Abruption)
- Stillbirth

**LOW-RISK**

Young women with mild hypotension

Women ≥ 30 yo with caesarean (without risk factor)

Women ≥ 30 yo with mild to moderate anaemia

**RISK UNLIKELY – VERY LOW RISK**

Young women without risk factor or possible mild anaemia

**HIGH-RISK & VERY HIGH-RISK**

Women ≥ 30 yo with hypotension and haemodynamic instability

Women ≥ 30 yo with severe anaemia

**INTERMEDIATE RISK**

Young women with hypotension and haemodynamic instability (without risk factor)

Young women with severe anaemia

Women ≥ 30 yo with moderate anaemia or risk factors

10
