# Supplementary material for: Early assessment of maternal bleeding: development and validation of a prognostic model predicting death and near misss
Source: Lancet Glob Health. Author manuscript; Available in PMC 2026 Oct 30. (PMC7619660; doi:10.1016/S2214-109X(25)00488-7)
Supplement: Supplementary file 2 [file EMS218566-supplement-The_Antifibrinolytics_Trialists_Collaborators_Obstetric_Group.docx]

The Antifibrinolytics Trialists Collaborators Obstetric Group

| **First name** | **Surname** |
| --- | --- |
| Francois-Xavier | Ageron |
| Homa | Ahmadzia |
| Monica | Arribas |
| Eni | Balogun |
| Amy | Brenner |
| Rizwana | Chaudhri |
| Catherine | Deneux-Tharaux |
| Amber | Geer |
| Katharine | Ker |
| Hugo | Madar |
| Raoul | Mansukhani |
| Oladapo | Olayemi |
| Luis D | Pacheco |
| Danielle | Prowse |
| George | Saade |
| Ian | Roberts |
| Haleema | Shakur-Still |
